# Nutrition in Sepsis: A Bench-to-Bedside Review

**DOI:** 10.3390/nu12020395

**Published:** 2020-02-02

**Authors:** Elisabeth De Waele, Manu L.N.G. Malbrain, Herbert Spapen

**Affiliations:** 1 Department of Intensive Care Medicine, University Hospital Brussels (UZB), 1090 Jette, Belgiumherbert.spapen@uzbrussel.be (H.S.); 2 Department of Nutrition, UZ Brussel, University Hospital Brussels (UZB), 1090 Jette, Belgium; 3 Faculty of Medicine and Pharmacy, Vrije Unversiteit Brussel (VUB), 1090 Brussels, Belgium

**Keywords:** nutrition, sepsis

## Abstract

Nutrition therapy in sepsis is challenging and differs from the standard feeding approach in critically ill patients. The dysregulated host response caused by infection induces progressive physiologic alterations, which may limit metabolic capacity by impairing mitochondrial function. Hence, early artificial nutrition should be ramped-up and emphasis laid on the post-acute phase of critical illness. Caloric dosing is ideally guided by indirect calorimetry, and endogenous energy production should be considered. Proteins should initially be delivered at low volume and progressively increased to 1.3 g/kg/day following shock symptoms wane. Both the enteral and parenteral route can be (simultaneously) used to cover caloric and protein targets. Regarding pharmaconutrition, a low dose glutamine seems appropriate in patients receiving parenteral nutrition. Supplementing arginine or selenium is not recommended. High-dose vitamin C administration may offer substantial benefit, but actual evidence is too limited for advocating its routine use in sepsis. Omega-3 polyunsaturated fatty acids to modulate metabolic processes can be safely used, but non-inferiority to other intravenous lipid emulsions remains unproven in septic patients. Nutrition stewardship, defined as the whole of interventions to optimize nutritional approach and treatment, should be pursued in all septic patients but may be difficult to accomplish within a context of profoundly altered cellular metabolic processes and organ dysfunction caused by time-bound excessive inflammation and/or immune suppression. This review aims to provide an overview and practical recommendations of all aspects of nutritional therapy in the setting of sepsis.

## 1. Introduction

Sepsis is still an ill-defined disease characterized by complex and time-dependent pathophysiologic processes. Despite remarkable improvement in diagnosis, monitoring, and treatment, mortality remains high [1,2]. Providing adequate nutrition in patients with sepsis or septic shock is challenging. This review focuses on different aspects of nutritional therapy in sepsis with specific focus on pathophysiology, caloric and protein dosing, feeding access, and pharmaconutrition.

### 1.1. Pathophysiology of Sepsis: A Two-Faced Immune-Inflammatory State

Sepsis is defined as a dysregulated host response caused by infection and associated with profound regional, microvascular, hemodynamic, metabolic, endocrine, and immune abnormalities that cause life-threatening organ dysfunction. Septic shock is a subset of sepsis accentuated by hypotension or an elevated serum lactate level despite adequate fluid resuscitation. Any reference to severe sepsis, a term formerly used interchangeably with sepsis, has been abandoned and the focus is turned more on assessment of organ failure than on identifying signs of inflammation [3]. Sepsis-induced multi-organ dysfunction is a clinical process characterized by progressive physiologic alterations in individual organs. Organ dysfunction may vary from mild to severe or irreversible. The cardiovascular and respiratory systems are most commonly affected. Lung failure classically manifests as acute respiratory distress syndrome (ARDS).

From a pathophysiological perspective, sepsis has often been considered as a syndrome evolving from an initial state of systemic and hypermetabolic inflammation into a more protracted phase of immunosuppression, characterized by lymphocyte exhaustion and apoptosis, diminished capacity of monocytes and macrophages to release pro-inflammatory cytokines, and occurrence of secondary infections [1]. Newer paradigms, however, suggest that the immune system spans two opposite directions by displaying signs of both excessive inflammation and immune suppression. Cellular metabolic processes undergo fundamental changes without return to normal homeostasis [2]. The extent of the inflammation and immune suppression varies between individuals and is determined by both hosts- (genetic heterogeneity, age, and comorbidities), pathogen- (load, type, and virulence), and therapy-related (timing, adequacy) factors [4]. Recently, this has also been referred to as cytokine storm or an imbalance between Damage Associated Molecular Patterns (DAMPS) and Pathogen Associated Molecular Patterns (PAMPS) [5]. Cellular and subsequent organ damage results from complex and interacting assaults involving endothelial activation, coagulopathy, disturbances in microcirculation, impaired mitochondrial function, enhanced apoptosis, increased gut permeability, and altered glucose and protein metabolism [5]. The intensity of the host response may also change in parallel with the clinical course. A significant number of patients who survive the initial septic insult endure a prolonged and complicated Intensive Care Unit (ICU) stay. This pathological state is typically associated with ongoing protein catabolism with loss of muscle mass, persistent organ failure, neuromuscular weakness, cachexia, poor wound healing, recurrent infections, and cognitive decline, and has been defined as “persistent inflammation-immunosuppression and catabolism syndrome” (PICS) [6]. Many PICS patients do not recover functional independence, remain institutionalized, and have poor long-term survival [7]. Poor or incomplete source control may additionally lead to a state of ongoing capillary leak and fluid accumulation, referred to as GIPS (global increased permeability syndrome) [8].

The metabolic response during acute sepsis weighs heavily upon the patient’s nutritional resources, requiring adequate nutritional intake, and generating large amounts of cellular waste products. Supplementing nutrients during the acute phase often must give way to life-saving resuscitation procedures. An approach favoring early enteral nutrition could expose patients with shock to gastrointestinal discomfort (vomiting) or harm (aspiration, gut ischemia) [9]. In contrast, patients in the PICS phase may better recover when receiving optimal caloric and protein therapy, supplemented with adequate doses of immune adjuvants, vitamins, and minerals. Ideally, nutritional intake should be patient-tailored and based on careful assessment of energy and protein needs.

The current review intends to update current knowledge on feeding the septic patient focusing on energy and protein requirements, feeding route, and the role of pharmaconutrition. The recommendations for nutrition therapy in sepsis are summarized in Table 1.

### 1.2. Calories and Proteins

#### 1.2.1. Calories

Caloric targeting in critically ill patients most often depends on predictive equations. However, a formula-based nutritional approach has no more value than flipping a coin and inevitably leads to under- or overestimation of calorie needs [10,11]. A worldwide audit in 10,000 patients from 880 ICUs showed poor adherence to nutrition guidelines. Caloric support was built up too slowly and not guided by weight or disease, leaving the majority of patients underfed [12]. Experts concur that caloric goals should be individualized and require an exact estimate of resting energy expenditure (REE) performed with indirect calorimetry (IC) as the gold standard [13]. Overall, bedside use of IC remains limited due to a lack of device standardization, erroneous results due to system leaks, and high inspired oxygen levels during mechanical ventilation, and logistic challenges (e.g., the need to repeat measurements over time). Groundbreaking work of the International Multicentric Study Group for Indirect Calorimetry (ICALIC) international study group intends to facilitate a more widespread clinical use of IC in the future [13]. Any caloric plan must also account for less controllable factors. For example, endogenous glucose production may cover up to 75% of energy needs during the first 3 to 4 days of critical illness [14]. This explains why caloric supplies should be built up slowly [15]. Hyper- and hypoglycemia are correlated with mortality [16], therefore close monitoring of glycemia is indispensable (crucial?).

The belief that sepsis is always associated with an acute hypermetabolic state has been challenged. Old IC data indeed demonstrated a steady increase of REE after sepsis onset, reaching a maximum of 1.7 times the baseline metabolism during the second week of ICU stay [17]. However, Kao et al. identified both hypo- and hypermetabolic activity in a small cohort of patients with sepsis and septic shock [18].

Insufficient nutrition and immune dysfunction had no synergistic effect on mortality in critically ill septic patients [19]. As expected, a well-fed patient with normal immune function had the best chance to survive. However, underfeeding patients with a marked immune disturbance did not prove harmful, whereas underfed immunocompetent patients had the worst prognosis. The Nutrition Risk in Critically ill (NUTRIC) score could not differentiate among these patients [20].

Large studies evaluating the outcome of septic patients subjected to different caloric regimens are not available. Zusman et al. [21] retrospectively investigated caloric and protein consumption in 1171 critically ill patients (77.2% on vasopressors, 22.8% sepsis). REE was measured with IC in all patients. Outcome was related to the percent of Administered Calories divided by REE (% AdCal/REE). This study corroborated that both under- and overnutrition were harmful. An AdCal/REE above 70% suggested an increase in mortality and was associated with increased duration of ventilation and length of ICU stay [21]. The TARGET trial evaluated the effect of delivering two different calorie levels (1.5 kcal/mL vs. 1.0 kcal/mL) for up to 28 days in mechanically ventilated ICU patients. Sepsis, defined according to the recent Sepsis-3 criteria, was present in 25% of patients in each treatment group. The target rate for both groups was 1 mL/kg/h, based on calculated ideal body weight with a maximum rate of 100 mL/h to minimize the risk of overfeeding. Patients in the energy-dense calorie group received a mean (± SD) of 1863 ± 478 kcal/day as compared with 1262 ± 313 kcal/day in the 1.0-kcal group. Higher calorie delivery did not affect mortality, receipt of or liberation from organ support, or incidence of infective complications [22].

Take-Home Message/Recommendations

- Measurement of REE with IC.
- A slow but steady increase of caloric load to reach target values when shock is resolved.

#### 1.2.2. Proteins

Enteral or parenteral protein substitution in critically ill patients mainly intends to ensure and enhance muscle protein synthesis in order to avoid or attenuate muscle wasting and to boost the neuromuscular revalidation process. However, optimal protein intake during critical illness is a topic of intense debate. Dose, timing, and risk-to-benefit ratio of protein supplementation during the different phases of sepsis are largely unexplored [23].

Weijs et al. [24] prospectively studied 843 critically ill patients and found an overall mortality benefit of early high protein intake in non-septic subjects only. More recent studies in medical ICU patients reported a positive correlation between early [25] or high [26] protein intake and decreased odds of mortality. However, both studies included few (respectively 17% and 21%) septic patients. Koekkoek et al. found a time-dependent effect of protein intake on outcome in ICU patients. They retrospectively studied the effect of a low (<0.8 g/kg/day), intermediate (0.8–1.2 g/kg/day), or high (>1.2 g/kg/day) protein diet in 455 medico-surgical patients ventilated for at least one week. Overall, the low protein group had the highest ICU, in-hospital, and 6-month mortality. High protein intake during the first 3 to 5 days also increased long-term mortality. Lowest 6-month mortality was observed when protein input gradually increased from low values during the first days of ICU stay to high levels from day 5 [27]. Prospective studies in representative patient cohorts, such as the EFFORT trial, which aims to resolve the high vs. low protein dose controversy [28], must be encouraged.

Take-Home Message

- We recommend administration of 0.8 g/kg/day during sepsis and a gradual increase up to 1.3 g/kg/day when shock resolves.

#### 1.2.3. Glucose

Optimal target blood glucose levels are not known. Hyperglycemia is associated with an adverse outcome and must be avoided. Tight glucose control is feasible in patients receiving parenteral nutrition if a strict protocol is followed [29].

#### 1.2.4. Lipids

Fatty acids are an important source of energy by their dense calorie content and contribute to the physical properties of the cell by their integration into cell membranes. They also play a role as precursors of bioactive lipid metabolites, such as prostaglandins, and are intertwined with cell response by gene expression [30]. Different sources of (intravenous) nutrition provide lipids and should be delivered daily to critically ill patients [31].

### 1.3. Enteral vs. Parenteral Feeding

Enteral nutrition (EN) is less costly than parenteral nutrition (PN) and theoretically represents the most physiological way of feeding. EN improves gastrointestinal blood flow, preserves the intestinal mucosal structure, stimulates enzymatic processes, and enhances the systemic immune response. An immediate clinical benefit of EN in septic patients could be the prevention of bacterial translocation and stress ulcerations. EN is considered to be safer than PN because no central venous access is required, and undesirable effects of PN (hyperglycemia, hyperlipidemia, fatty liver, etc.) are avoided. However, EN is not without drawbacks. Feeding may be suboptimal due to irregular absorption. Gastric content may not pass beyond the pylorus (slow gastric emptying, ileus, etc.) which increases the aspiration risk. EN may also be hampered by transit problems (vomiting, diarrhea) and is contraindicated when the gut is ischemic, injured, or obstructed [32]. In shocked patients, EN may increase the risk of bowel ischemia by imposing too much digestion workload on a hypo-perfused gut [33].

A meta-analysis of 16 randomized controlled trials (RCTs) enrolling 3225 critically ill patients showed that starting EN within 24 h of ICU admission did not reduce mortality. In a priori identified subgroups, early EN increased survival, compared with delayed enteral intake, whereas no mortality difference was observed between early EN and PN. Patients receiving early EN were also less likely to develop pneumonia [34]. The EDEN trial randomized patients with acute lung injury within 48 h of ICU admission to receive either low-volume (trophic) or full EN for the first week. There were no differences in ventilator-free days, infectious complications, or mortality between groups, but trophic EN caused less gastrointestinal intolerance [35].

The much-debated Early Parenteral Nutrition Completing Enteral Nutrition in Adult Critically Ill Patients (EPaNIC) trial included ICU (mainly cardiac surgery and approximately 20% septic) patients at risk for malnutrition, despite receiving early enteral feeding, plus micronutrients, within a tight glycemic control protocol. Late, as compared with early initiation of PN, was associated with lower mortality, less need for ventilation and renal support, shorter ICU and hospital stay, and fewer infectious complications [36]. A post hoc analysis of this trial suggested that the highest amount of nutritional intake was associated with a higher infection rate and a delay in recovery [37]. Of note is that the dosing of nutrition in the EPaNIC trial was determined by calculating the metabolic rate. Poor correlation of this formula-based approach with measured energy expenditure may have contributed to the observed negative effect of early PN on outcome [38]. A recent Cochrane review compared the effects of EN versus PN or a combination of EN and PN in adult ICU (in particular trauma, emergency, and postsurgical) patients. Twenty-five randomized and quasi-randomized studies with 8816 participants were included for analysis. The evidence to determine whether EN is better or worse than PN or EN plus PN for mortality, number of ventilator-free days and adverse events were largely insufficient. EN was associated with less sepsis than PN [39].

Only two large RCTs have specifically assessed the feeding route in critically ill patients. The multicenter CALORIES trial randomly assigned patients to either a parenteral (*n* = 1188) or enteral (*n* = 1195) feeding route. Nutrition was delivered within 36 h after admission and continued for up to 5 days. The number of septic patients was not specified but more than 80% of subjects in both study arms received “vasoactive” medication. Caloric intake was similar in both study groups, but the majority of patients did not reach the target value of 25 kcal/kg/day. The 30-day mortality was not different between groups (33.1% vs. 34.2%: parenteral vs. enteral; *p* = 0.57). There was no difference in incidence of infections and a non-significant trend toward an increased risk of gastrointestinal complications with enteral feeding [40].

The multicenter NUTRIREA-2 trial randomly assigned 2410 ICU patients receiving invasive mechanical ventilation and vasopressor support to either PN or EN. Nutritional support was started as soon as possible after randomization and no later than 24 h after intubation. Approximately two-thirds of patients in both groups were classified as septic shock. Parenterally fed patients were switched to enteral feeding when they met predefined criteria for entry into the recovery phase, which reflected nutritional adaptation to the course of acute critical illness. In both groups, caloric amounts approximated the 20 kcal/kg/day target. Between-group differences in calorie and protein intakes were very small. By day 28, 37% of the patients in the enteral group, and 35% of the patients in the parenteral group had died (*p* = 0.33). Both feeding groups had a similar cumulative incidence of ICU-acquired infections but the enteral cohort had higher cumulative incidences of digestive inconveniences and complications (vomiting, diarrhea, bowel ischemia, and acute colonic pseudo-obstruction) [41].

In conclusion, the Cochrane analysis and two specifically conceived large RCTs do not show the superiority of the enteral over the parenteral route for early nutritional support in critically ill patients. However, the NUTRIREA-2 trial demonstrates a relationship between early EN and gastrointestinal complications in patients with shock. This indicates that full EN feeding should be avoided during the acute phase of septic shock. PN may be a safer route in these patients. The appropriate time to start PN remains a matter of discussion. However, supplemental PN should be considered in any patient at risk of undernutrition if EN fails to reach calorie targets after 3 to 7 days [31]. Uncontrolled shock, uncontrolled hypoxemia and acidosis remain contraindications for early enteral nutrition in the European Society Intensive Care Medicine ESICM practice guideline [9].

Take-Home Messages

- EN has no proven clinical benefit over PN and is associated with more digestive disorders. However, when the gut works, we recommend using it.
- Early EN in combination with supplemental PN is the preferred procedure to reach at least 80% of caloric needs by day 3.

### 1.4. Pharmaco-Nutrition

Pharmaco-nutrition refers to the addition of nutrients with specific beneficial actions (e.g., antioxidant effects) to standard feeding, and specifically aims to invigorate gut mucosal and systemic immune defense mechanisms, and to shackle an excessive pro-inflammatory response during the catabolic phase of illness [42,43]. The most relevant “pharmaco-nutrients” in septic patients are the amino-acids glutamine and arginine, omega-3 fatty acids, selenium, and vitamin C.

#### 1.4.1. Glutamine

Glutamine is an essential nutrient for enterocytes and immune cells. Glutamine maintains gut barrier function, exerts antioxidant and cytoprotective effects, stimulates nucleotide synthesis, preserves neutrophil bacterial killing, and enhances lymphocyte and macrophage proliferation and secretion. Intense immune activity and/or hyper catabolism, as occurring in burn injury, trauma, and sepsis, are associated with increased glutamine consumption and a dramatic fall in plasma glutamine concentrations. The resulting loss in glutamine-sustained processes will cause severe impairment of immune function. Hypoglutaminemia is an independent predictor of mortality and/or poor clinical outcome in critically ill patients [44,45].

The Scottish Intensive care Glutamine or selenium Evaluative Trial randomized 502 critically ill patients to receive PN supplemented with glutamine (20.2 g/day), selenium (500 µg/day), or both, for up to seven days. Among the 250 patients who received any glutamine, 60% were identified with sepsis. Adding glutamine to parenteral feeding had no effect on mortality, the occurrence of new infections, days of antibiotic use, organ failure, and length of stay [46].

The multicenter Scandinavian glutamine trial investigated intravenous glutamine supplementation (0.283 g/kg body weight/12 h) in 413 adequately fed ICU patients. The number of septic patients was not specified. The glutamine group had no change in organ failure scores, and a lower ICU mortality, which was not sustained at 6 months [47].

In the REDOXS trial, Heyland et al. randomly assigned 1223 mechanically ventilated critically ill patients with multi-organ failure to receive early intravenous and enteral supplements of glutamine, antioxidants, both, or placebo. Approximately one-third of the 611 patients who received glutamine was diagnosed with sepsis. Glutamine had no effect on infectious complications or organ failure rate. On the contrary, 6-month and in-hospital mortality were significantly higher in glutamine-treated subjects [48]. A post hoc analysis by the same group confirmed the harmful effect of both glutamine and antioxidants, particularly in patients with renal dysfunction [49].

The multicenter MetaPlus trial included 301 mechanically ventilated ICU patients. Subjects were randomized to receive either an experimental tube feed enriched with glutamine, omega-3 fatty acids, and antioxidants (21% patients with sepsis) or standard high-protein EN (23% patients with sepsis). Feeding was initiated within 48 h after ICU admission and continued for a maximum of 28 days. There were no statistically significant differences in primary (incidence of infectious complications) or other (duration of mechanical ventilation, ICU or hospital length of stay) endpoints except for a higher 6-month adjusted mortality rate in medical patients treated with immune-modulating nutrients: 54% vs. 35% (*p* = 0.04) [50]. Low dose glutamine (<0.35 g/kg/day intravenously or <0.5 g/kg/day enterally) to complete amino acid content can be safely administered to patients receiving PN [51].

Take-Home Message

- Glutamine administration has no benefit and may even be harmful in septic patients.

#### 1.4.2. Arginine

Sepsis is accompanied by enhanced consumption, impaired synthesis, and a decreased supply of the semi-essential amino-acid arginine [52]. This arginine-deficient state impairs immune homeostasis and increases the risk of nosocomial infections. Accordingly, supplementation of L-arginine is thought to contribute in restoring physiologic processes in septic patients, including protein synthesis, organ perfusion, and wound healing.

Arginine supplementation in septic patients has transient hemodynamic side effects when supplied as a bolus that is not seen when supplied continuously [53]. In a small cohort of septic shock patients, intravenous arginine infusion increased nitric oxide production and reduced whole-body protein breakdown without altering hemodynamic parameters [54]. Bertolini et al. showed that an enteral diet composed of extra L-arginine, omega-3 (ω-3) fatty acids, and antioxidants, compared to PN, was associated with excess mortality in patients with severe sepsis [55]. Supradietary doses of parenteral L-arginine increased shock severity, organ injury, and mortality in a canine peritonitis model [56]. High arginine plus fish oil formulas compared to sole fish oil and/or arginine formulas have differing effects on mortality in critically ill patients suggesting that arginine may counteract the benefits of fish oil [57].

Take-Home Message

- Lack of firm evidence argues against arginine supplementation in sepsis and septic shock.

#### 1.4.3. Omega-3 Fatty Acids

Fish oil contains the ω-3 polyunsaturated fatty acids (ω-3 PUFAs), eicosapentaenoic acid (EPA), and docosahexaenoic acid (DHA). EPA and DHA modulate loco-regional and distant metabolic processes and reduce inflammation [58]. Early studies investigating enteral ω-3 supplementation in patients with sepsis and septic shock yielded divergent results, with some reporting reduced mortality and fewer infections [59,60], whilst others did not find any survival benefit or decrease in infectious complications [61].

Pontes-Arruda et al. [62] observed less progression to severe sepsis and septic shock, less organ failure, shorter ICU and hospital stays, but no difference in 28-day all-cause mortality in patients fed with enteral nutrition enriched with EPA, γ-linolenic acid, and antioxidants, as compared with controls. However, the study included mainly elderly patients already receiving enteral feeding, which makes extrapolation of the results to a general septic population unreliable. A post hoc analysis of the MetaPlus trial found that the harmful effect of enteral feeding enriched with glutamine, ω-3 fatty acids, and antioxidants on mortality in the medical subgroup was probably mediated by an early increase in (EPA + DHA)/long-chain fatty acid plasma ratio [63].

A meta-analysis of 11 randomized controlled trials (RCTs) in 808 septic patients found no significant effect of ω-3 PUFAs on overall mortality or infectious complications. However, ω-3 PUFA recipients had a markedly reduced ventilation need [64]. This was corroborated by Lu et al. who analyzed 17 RCTs of enteral and parenteral ω-3 PUFA administration in 1239 patients diagnosed with sepsis or septic shock. Compared to no supplementation or placebo, ω-3 supplementation did not influence mortality but reduced ICU length of stay and duration of mechanical ventilation [65]. Of note is the large heterogeneity of the patient populations enrolled and the very low evidence supporting the benefit of ω-3 PUFAs on other outcomes besides mortality.

Correlation analysis of 18 RCTs of enteral and parenteral ω-3 PUFA administration in 1790 patients with similar severity of sepsis and sepsis-induced ARDS further underscored that ω-3 fatty acids had no positive effect on mortality [66].

Koekkoek et al. [67] reviewed 24 RCTs enrolling 3574 medical, surgical, and trauma patients that addressed relevant clinical outcomes of fish oil-containing EN in critically ill patients. Enteral fish oil supplementation reduced ICU length of stay and ventilation duration but did not affect 28-day, ICU or hospital mortality. However, 28-day mortality, ICU length of stay, and ventilation duration were significantly decreased in the subgroup of patients with ARDS. No significant effects on infectious complications were observed in overall or subgroup analyses [67].

Based on a meta-analysis of 7 RCTs (*n* = 955 patients), Zhu et al. concluded that enteral supplementation with ω-3 PUFAs in patients with ARDS seemed ineffective in terms of 28-day mortality, ventilation-free days, and ICU-free days [68]. Analyzing the same ARDS patient population, Santacruz Herrera et al. reported that enteral substitution including ω-3 PUFAs and competitive analogs of ω-6 PUFAs was associated with decreased mortality when the comparator solution contained a higher quantity of ω-6 PUFAs and a far greater amount of lipid than recommended in clinical practice [69]. In a recent meta-analysis, Langlois et al. specifically evaluated the clinical benefits of mainly enteral ω-3 PUFA administration compared with placebo in critically ill patients with ARDS. Twelve RCTs (*n* = 1280 patients) were reviewed and analyzed. ω-3 PUFA administration, particularly when given as a continuous infusion in combination with γ-linolenic acid and antioxidants, was associated with significant early and late improvement of oxygenation. Patients who received ω-3 PUFAs tended to have a reduced duration of mechanical ventilation and ICU stay. Infectious complications remained unchanged. Only trials published before 2011 showed a significant reduction in mortality, which might not relate to feeding but to a change in therapeutic approach of ARDS [70]. Variables unrelated to nutrition, such as sepsis stage or severity and novel treatment strategies in ARDS, may have influenced study results.

Take-Home Messages

- The scientific evidence to justify fish oil supplementation in patients with sepsis or septic shock is weak.
- Administering ω-3 PUFAs might improve gas exchange and subsequent weaning from mechanical ventilation in ARDS patients, but it remains unclear whether this therapeutic effect depends on the feeding route (enteral vs. parenteral), method (bolus vs. continuous infusion), or composition (lipid type and amount).

#### 1.4.4. Selenium

Selenium is a component of selenoproteins with antioxidant, anti-inflammatory, and immunomodulatory properties. Low selenium concentrations in patients with systemic inflammation or sepsis are associated with defective neutrophil and macrophage function and a reduced antioxidant defense [71].

Patients included in the REDOXS trial received enteral antioxidant therapy, including 300 µg selenium and a total selenite dose of 800 µg. There was no effect on clinical outcome. Three meta-analyses showed a beneficial effect of selenium supplementation on mortality in septic patients [72,73,74]. However, estimates were very imprecise and the positive effect was associated with high-dose selenium only. Subsequent RCTs investigating high-dose parenteral selenium administration in septic patients showed no beneficial effect on mortality [75,76].

The most recent meta-analysis, including 13 RCTs comparing intravenous selenium and placebo in septic patients, failed to detect an association of selenium treatment with decreased mortality. Selenium supplementation was associated with earlier shock reversal and less ventilator-associated pneumonia, but did not decrease the incidence of renal failure, secondary infection, or duration of mechanical ventilation [77].

The treatment effect of selenium may be dependent on the dose, the route of administration, combination with other nutrients, and the patient population studied.

Take-Home Message

- Selenium alone or in combination with other antioxidants is not recommended in sepsis.

#### 1.4.5. Vitamin C

Vitamin C has important vascular protective effects by inhibiting oxidative stress, modulating intracellular signaling pathways, and maintaining homeostatic levels of nitric oxide [78]. Vitamin C also is an essential cofactor for endogenous production of norepinephrine, epinephrine, and vasopressin [79]. Septic patients typically have very low or undetectable serum levels of vitamin C despite recommended enteral and parenteral intakes [80]. A recent meta-analysis enrolling 142 medical, surgical, burn, and trauma patients investigated the effects of isolated intravenous supplementation of vitamin C. Compared with controls, vitamin C recipients had less vasopressor and ventilation needs and a trend for better fluid tolerance. No difference in mortality rate was noted [81]. The one study in septic patients included in this meta-analysis showed a positive impact of vitamin C on the extent of organ failure, biomarkers of inflammation and endothelial injury [82]. The optimal vitamin C dose is still a topic of debate and dose-response studies are definitely needed. High-dose intravenous vitamin C is regarded as safe. Potential side-effects (oxalate nephropathy, pro-oxidant effects, and hypotension) are seldom. It is recommended to start vitamin C at a high dose for a short course (e.g., 4 days) during the acute phase of sepsis when oxidant stress is prominent. Thereafter, vitamin C can be continued at lower (nutritional) doses to allow generation of low concentrations of reactive oxygen species, which are essential for physiological signaling and repair [83].

Other studies found that the use of high-dose vitamin C in patients with severe burns resulted in less positive fluid balance and less secondary abdominal hypertension [84]. However, these results should be interpreted with caution as vitamin C may have resulted in increased osmotic diuresis explaining the beneficial results.

Marik et al. retrospectively studied 47 septic patients before and 47 patients after treatment with vitamin C 1.5 g q6 h for 4 days, thiamine 200 mg q12 h for 4 days, and hydrocortisone 50 mg q6 h for 7 days. Hospital mortality was 8.5% in the treatment group compared with 40.4% in the control group (*p* < 0.001) [85]. This remarkable result must be interpreted with caution, considering the small number of patients, the retrospective study design, and the biological implausibility of such a strong treatment effect. Many randomized controlled trials within the context of sepsis, septic shock, and acute lung injury are forthcoming to investigate the potential beneficial effect of vitamin C alone and in combination with hydrocortisone and thiamine.

Take-Home Message

- At present, insufficient evidence supports routine vitamin C administration in sepsis.

### 1.5. Nutrition Stewardship

Nutrition stewardship is defined, in analogue with fluid stewardship, as a series of coordinated interventions, introduced to select the optimal type of nutrition, dose and duration of therapy that results in the best clinical outcome, prevention of adverse events, and cost reduction [86]. This can be accomplished by adhering to the 6 D’s (diagnosis—drug—dose—duration—de-escalation—discharge) [8].

#### 1.5.1. Diagnosis

Correct nutrition therapy starts with an adequate assessment of the patient’s nutritional status [87] and metabolic evaluation via indirect calorimetry in combination with other monitoring tools, such as BIA and nitrogen balance [31].

#### 1.5.2. Drug

Critical care physicians should consider nutrition as drugs that have indications and contraindications, and potential adverse effects, and pay particular attention to the different compounds and their specificities (calories, nitrogen, protein, glucose, lipids, and micronutrients). For each type of nutrition, there are distinct indications and specific side effects [88].

#### 1.5.3. Dose

“Sola dosis facit venenum” or “the dose makes the poison”. As discussed above, there are various important considerations for nutritional prescription, as calorie and protein dosing are correlated with mortality [21], and pharmacokinetics and dynamics need to be taken into account, as well as volume kinetics, since nutrition may also contribute to fluid accumulation [8].

#### 1.5.4. Duration

The duration of total or supplemental artificial nutritional therapy is equally important and parenteral nutrition must be tapered when shock is resolved and the gastrointestinal tract is normally functioning [9].

#### 1.5.5. De-escalation

The final step in artificial EN or PN nutrition therapy is to consider withholding or withdrawing when they are no longer required.

#### 1.5.6. Discharge

Correct (dis)continuation or tapering of artificial nutritional therapy and (when needed and indicated) prescription post-discharge from ICU, or hospital, is part of the nutritional care plan and should meet quality standards [89].

## 2. Conclusions

Nutritional assessment and treatment in sepsis is cumbersome because the proportion of septic patients enrolled in ICU nutrition trials is low and sepsis-related inflammation, metabolic changes, immune reactivity, and organ dysfunction all determine or compromise the feeding process.

Prescribing calories and proteins in septic patients is challenging. Patients may be hypo- or hypermetabolic and energy needs change considerably over time and in between patients. Calculating energy requirements frequently underestimates real needs and exposes to underfeeding. Scarce evidence suggests that higher calorie delivery does not affect morbidity and mortality. Caloric goals ideally are individualized by measuring resting energy expenditure with indirect calorimetry, which has become practical and feasible. Nutritional screening at different stages of the septic process likely is more appropriate than a single “bullseye” or a quotidian approach.

Dose, timing, and the risk-to-benefit ratio of protein supplementation in sepsis are unexplored. Preliminary evidence suggests that early high-dose protein supplementation should be avoided.

Both the enteral and parenteral route is useful to feed septic patients. Apart from limitations and drawbacks specific to each particular route, early EN increases the risk of severe digestive complications in shock, whereas early non-individualized PN is associated with worse ICU outcomes. PN remains a valuable option if EN fails to reach calorie targets after 3 to 7 days.

The theoretical benefit of adjuvant pharmaco-nutrition does not translate into better outcome in patients with sepsis or septic shock. Clinical evidence to justify the use of glutamine, arginine, selenium, or fish oil is weak and excessive supplementation may be harmful. Omega-3 fatty acid supplementation is associated with improved outcome of ARDS but it remains unclear whether this effect depends on the component itself or on improved therapeutic management of ARDS. Over a dozen RCTs are actually assessing the effects of intravenous administration of high-dose vitamin C in monotherapy, or combined with thiamine and hydrocortisone in critically ill patients, particularly those with sepsis. It is time to consider nutrition therapy as any other drug and to implement nutrition stewardship in your ICU following the 6 D’s: diagnosis—drug—dose—duration—de-escalation—discharge. Current knowledge can guide nutrition therapy in sepsis (Table 1).

**Figure 1:**
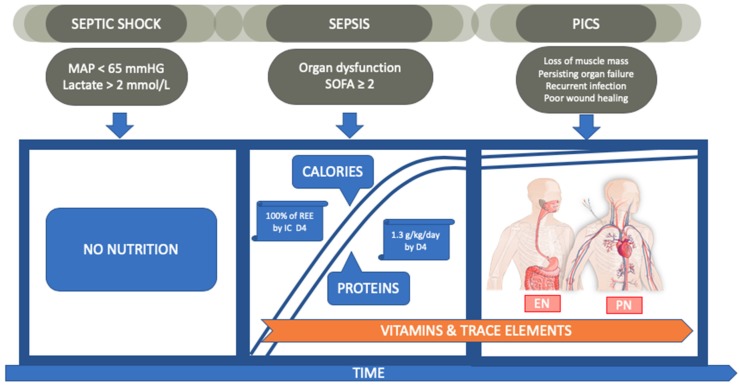
Flow Visual representation of nutritional therapy in sepsis. Mean Arterial Pressure (MAP); Sequential Organ Failure Assessment (SOFA); Post Intensive Care Syndrome (PICS); resting energy expenditure (REE); indirect calorimetry (IC); Day 4 (D4); enteral nutrition (EN); parenteral nutrition (PN).

As noted in Figure 1, Nutrition for patients in sepsis should include the provision, by enteral and/or parenteral route, of calories guided by indirect calorimetry and proteins at 1.3 g/kg/day after shock symptoms wane, in combination with low dose glutamine in parenteral only fed patients, and including carbohydrates, proteins, and lipids.

## Figures and Tables

**Table 1:** Nutritional recommendations in sepsis.

Nutrient	Recommended Dose
Caloric needs	Determined by indirect calorimetry
Protein	0.8–1.3 g/kg/day
Lipids	0.7–1.5 g/kg/day
Glucose	1–1.5 g/kg/day
Glutamine	<0.35 g/kg/day IV or <0.5 g/kg/day enterally in TPN fed patients
Fluid	1 mL/kg/h

TPN: Total Parenteral Nutrition.
